# Novel MEN 1 gene findings in rare sporadic insulinoma—a case control study

**DOI:** 10.1186/s12902-015-0041-2

**Published:** 2015-08-26

**Authors:** Viveka P. Jyotsna, Ekta Malik, Shweta Birla, Arundhati Sharma

**Affiliations:** Department of Endocrinology and metabolism, All India Institute of Medical Sciences, Room No. 305, Third Floor, Biotechnology Building, New Delhi, India; Department of Anatomy, All India Institute of Medical Sciences, New Delhi, India

## Abstract

**Background:**

Insulinomas, which are rare tumors causing hyperinsulinemic hypoglycemia are usually sporadic but may also occur in association with multiple endocrine neoplasia type 1 (MEN-1) syndrome an autosomal dominant disorder caused by MEN1 gene mutations. MEN1 encodes a nuclear protein Menin, a tumor suppressor which acts as an adapter and interacts with partner proteins involved in crucial activities like transcriptional regulation, cell division, proliferation and genome stability.

This study reports on clinical findings and mutation screening in sporadic insulinoma patients.

**Methods:**

Seventeen patients diagnosed with insulinoma were recruited along with 30 healthy volunteers who acted as controls for the present study. The patients presented with symptoms of sweating, tremors, drowsiness, palpitations, loss of consciousness, abnormal behavior, seizures and weight gain. Detailed clinical and family history was collected from all the participants along with 5 ml of blood sample after taking informed consent.

Genomic DNA isolated from blood was subjected to MEN1 gene amplification followed by direct sequencing. Nucleotide sequences obtained were compared with published MEN1 cDNA sequences. Prediction of functional effects of novel changes was done using various bioinformatics algorithms.

**Results:**

Molecular analysis revealed presence of three novel exonic mutations (M561K, Q192K and Q261Q), two novel intronic variations c.445-44G → A and c.913-42G → C in introns two and six respectively and three reported exon SNPs; H433H (rs540012), D418D (rs2071313), A541T (rs2959656) and one intronic SNP (rs669976).

**Conclusions:**

The study identified presence of novel pathogenic MEN1 mutations in sporadic cases of insulinoma. The new mutations identified were in regions involved in defective binding of menin to proteins implicated in genetic and epigenetic mechanisms. The outcome of the study extends the growing list of MEN1 pathogenic mutations even in sporadic cases providing consequential insight into phenotypic heterogeneity and in the expression of individual mutations.

## Background

Insulinomas are neuroendocrine tumors of the pancreas with an incidence of 0.4 % [1] and present clinically with hypoglycemia [2]. They are usually benign solitary tumours [3] but 5–12 % of the cases have distant metastasis at diagnosis. Most of the insulinomas arise sporadically, but 5–8 % may develop as part of the hereditary multiple endocrine neoplasia type 1 (MEN1) syndrome [4, 5]. Insulinoma is the second most common hormone secreting pancreatic neuroendocrine tumor in MEN1. Other pancreatic neuroendocrine tumors are gastriomas, glucagonomas, vasoactive intestinal polypeptidomas and their occurrence is 40–70 % in MEN1 patients [6]. Most of the insulinomas are small in size (<2 cm) and over 99 % of them originate in the pancreas with rare cases derived from ectopic pancreatic tissue [7]. Surgical removal is the main treatment and the neurological sequelae of hypoglycemia after surgery have been shown to revert [8].

**Table 1:** Tumor characteristics and MEN1variations in insulinoma patients

ID.	Age/Sex	Tumour characteristics			MEN1 variations found
		Location	Size	Nature	
M1	35/M	3	2.0 ^a^2.0 cm	B	Q260R^a^
					A541T (rs2959656)^d^
					H433H (rs 540012)^d^
M2	38/M	3	4.8^a^5.9 cm	M	H433H (rs 540012)^d^
M3	23/M	3	1.0^a^0.8 cm	B	H433H (rs 540012)^d^
					D418D (rs2071313)^d^
M4	34/M	3	0.8^a^0.9 cm	B	H433H (rs 540012)^d^
					A541T (rs2959656)^d^
M5	42/M	3	1.0 x 0.8 cm	B	H433H (rs 540012)^d^
					D418D (rs2071313)^d^
M6	35/F	2	2.0 ^a^2.0 cm	B	Q260R^a^
					H433H (rs 540012)^d^
					D418D (rs2071313)^d^
					A541T (rs2959656)^d^
M7	65/F	2	1.5^a^1.2 cm	B	M561K^a^
					H433H (rs 540012)^d^
					D418D (rs2071313)^d^
					A541T (rs2959656)^d^
					Intronic rs 669976 ^e^
M8	50/F	2	3.0^a^2.0 cm	B	H433H (rs 540012)^d^
					D418D (rs2071313)^d^
					A541T (rs2959656)^d^
M9	25/M	4	4.0^a^5.0 cm	B	H433H (rs 540012)^d^
					A541T (rs2959656)^d^
M10	29/F	4	1.0^a^0.5 cm	B	H433H (rs 540012)^d^
					A541T (rs2959656)^d^
M11	16/M	3	1.0 x 1.0 cm	B	H433H (rs 540012)^d^
					A541T (rs2959656)^d^
M12	18/F	3	2.0 x 1.0 cm	B	H433H (rs 540012)^d^
					Intronic rs 669976 ^e^
					A541T (rs2959656)^d^
M13	17/M	4.	2.0 ^a^2.0 cm	B	Q192K ^a^
					H433H (rs 540012)^d^
M14.	45/F	1	3.0^a^2.0 cm	B	Q261Q^b^
					H433H (rs 540012)^d^
					D418D (rs2071313)^d^
					A541T (rs2959656)^d^
M15.	46/M	4	1.0^a^1.0 cm	B	H433H (rs 540012)^d^
					A541T (rs2959656)^d^
M16.	30/M	4	1.0^a^1.0 cm	B	Intronic c.913-42G → C^c^
					H433H (rs 540012)^d^
					D418D (rs2071313)^d^
M17	41/F	4	0.85^a^0.65 cm	B	Intronic c.445-44G → A^c^
					H433H (rs 540012)^d^
					A541T (rs2959656)^d^

Legend: *M* male, *F* female, *B* benign, *M* malignant, 1-junction of neck and body of the pancreas, 2-uncinate process of the pancreas, 3-head of the pancreas, 4-tail of the pancreas. ^a^ - novel non synonymous exonic mutations, ^b^- novel synonymous exonic mutation, ^c^ novel intronic variations, ^d^ -reported exonic polymorphisms, ^e^- reported intronic polymorphisms

MEN 1(OMIM 613733) located on chromosome 11q13.1, consists of ten exons spanning a region of >9 Kb of genomic DNA [9] and encodes a 621 amino acid protein ‘menin’, a putative tumor suppressor gene associated with insulinoma [10]. Menin acts as an adapter and interacts with partner proteins involved in crucial activities like transcriptional regulation, cell division, proliferation and genome stability. Although located predominantly in the nucleus, in the non dividing cells it is found in the cytoplasm also [11]. We report here the results of MEN 1 screening in 17 sporadic insulinoma patients along with their clinical findings.

## Methods

The study protocol adhered to the tenets of the Declaration of Helsinki and was approved by the Institutional Ethics Committee of All India Institute of Medical Sciences, New Delhi. Written informed consent was obtained from each patient for publication of individual clinical details.

A total of 17 patients diagnosed with insulinoma from the department of Endocrinology and Metabolism and 30 healthy volunteers as controls were recruited for the present study. Insulinoma was suspected when a patient presented with recurrent hypoglycemia and a diagnosis of insulinoma was made when during spontaneous or fast induced hypoglycemia plasma glucose was <45 mg/dl, serum insulin was > 6 μU/ml and C peptide was > 2.5 ng/ml [2]. After a biochemical diagnosis of hyperinsulinomic hypoglycemia was confirmed, localization of insulinoma was done by various imaging modalities like multiphasic CT, MRI, arterial calcium stimulation and venous sampling, endoscopic ultrasound and intraoperative ultrasound. After localization, the tumor was removed and location and size of the tumor intraoperatively was noted. From all the participants detailed family history was noted and peripheral blood drawn in EDTA vials for DNA extraction.

### Mutation analysis

Genomic DNA was isolated from peripheral blood leukocytes using standard protocol and subjected to PCR amplification of all the MEN1 coding exons using 80–100 ng DNA, 1.25 mM MgCl2, 0.25 mM of each of the dNTPs (Invitrogen, Carlsbad, CA, USA), 20pM of each primer and 0.5 units of Taq Polymerase (Invitrogen, Carlsbad, CA, USA) in a 25 ul volume mixture using thermocycler ABI 9700 (Applied Biosystems, Foster City, CA).

All the amplified products were purified using Qiagen kits (Qiagen, GmbH, Hilden, Germany) and were then sequenced using ABI-3100 Genetic Analyzer (ABI).

Nucleotide sequences were compared with the published cDNA sequences of MEN1 gene (GenBank accession number ENST00000312049). Prediction of functional effects of the novel variations was done using algorithms Mutation Taster, SIFT and PROVEAN.

## Results

Out of 17 patients, ten were males and seven were females. Main presenting symptoms were recurrent hypoglycemia, seizures, confusion and excessive weight gain. History of sweating, tremors and drowsiness was present in all (100 %) the patients, 13 (76.47 %) patients had palpitations, weakness and weight gain whereas only 12 (70.59 %) patients had loss of consciousness, abnormal behavior and seizures. Out of all, 15 (88.24 %) patients had been to the neurologist and/or psychiatrist for treatment before a diagnosis of insulinoma was made. The mean age at presentation was 35 ± 13 years. The mean duration of symptoms was 3.56 years (range 1 to 15 years) before diagnosis of insulinoma was established. The mean levels of insulin was 17.36 μU/ml (range 6.5 to 153), C peptide was 5.05 ng/ml (range 0.88 to 11.90) and mean plasma glucose of 32.86 mg/dl (range 18 to 45 mg/dl).

Other components of MEN1 syndrome were ruled out by taking history of symptoms, noting sign and laboratory tests. Clinical features of anterior pituitary tumors asked for were irregular periods, galactorrhoea, and infertility in females, erectile dysfunction in males, headache, visual impairment, central obesity and proximal muscle weakness in both. Signs looked for were presence of violaceous stria, buffalo hump, acral enlargement, prognathism, frontal bossing, thyroid enlargement. Clinical features of hyperparathyroididm asked for were bone pain, history of fractures, renal stones and abdominal pain. Laboratory testes performed to rule out pituitary or parathyroid tumors were serum fasting prolactin, ACTH, cortisol, growth hormone and insulin like growth factor1, T4, TSH, intact PTH, calcium and phosphorus. These symptoms/signs were absent and tests were normal in all the patients.

There was no history of consanguinity in any of the families. Only one patient (6 %) had malignant insulinoma and rests were benign. Insulinomas were found located in different parts of the pancreas and the sites of the primary tumors were junction of neck and body in one patient (6 %), uncinate process in two patients (12 %) and in seven patients each (41 %) it was located in head and tail of the pancreas (Table 1). All the patients (100 %) underwent surgery as curative procedure. Only one patient underwent resection twice because of recurrence of the tumor at different times.

MEN1 screening revealed presence of novel changes which include two non-synonymous missense mutations; M561K in which a nucleotide changes from T to A resulted in amino acid methionine being replaced by lysine at codon 561 in exon ten and Q192K resulting due to sequence variation C > A leading to amino acid change from Glutamine to Lysine at codon 192.

A novel synonymous mutation (Q261Q) resulting in a nucleotide change from G to A at codon 261 was also identified with no change in the amino acid glutamine (Q).

Three reported exonic polymorphisms were identified: H433H (rs540012) in all patients and controls, D418D (rs2071313) in 8 (47 %) patients; none of the controls and A541T (rs2959656) in 11 (65 %) patients and in all the controls.

A reported intronic polymorphism rs669976 was identified in two (12 %) patients and was absent in all the controls. Two novel intronic variations c.445-44G → A in intron 2 and c.913-42G → C located in intron six were also identified (Table 1).

Assessment for pathogenicity using MutationTaster, PROVEAN and SIFT predicted all the novel exonic mutations and novel intronic variation (c.913-42G → C) to be deleterious and disease causing alterations.

To confirm the findings from genetic screening and bioinformatics analysis, 30 healthy controls were screened to check for the presence of the novel variations. The changes were not identified in any of them eliminating their possibility of being polymorphisms.

## Discussion

In the present study we report clinical and genetic findings of 17 patients diagnosed with insulinoma. Clinical presentations of insulinomas are diverse and heterogeneous. Hypoglycaemia results in various neuroglycopenic and adrenergic symptoms. Neuroglycopenic symptoms include many psychiatric and neurological manifestations like behavioural changes, confusion, agitation, blurred vision and finally seizures [12, 13]. All the 17 patients (100 %) in the present report had hypoglycemia with neurological manifestations like confusion and seizures.

Pancreatic islet cell tumors can be the first manifestation in 15 % of all the MEN1 cases [14]. Insulinoma may arise either due to loss of heterozygosity (LOH) of the chromosome region 11q13 or due to presence of various mutations in the MEN-1 gene [15, 16] in accordance with the Knudson’s hypothesis on cancer [17]. Most of the insulinomas are benign and are found located anywhere in the pancreas and surgical removal is the first line of treatment. Malignant insulinomas are comparatively rare and are found in around 5–12 % of reported insulinoma cases [18] . Without curative treatment MEN1 insulinomas are found to be associated with earlier mortality in the patients[19] 

In the present study, only one patient (6 %, *n* = 17; 38 years, male) had malignant insulinoma. The size of the tumor was 4.8*5.9 cm located in the head of the pancreas with multiple metastases in the liver. He was operated and died within two years of diagnosis. No MEN1 mutation was identified in this patient indicating possible presence of other important genetic events like involvement of other unidentified gene/s, chromosomal instability resulting in insulinoma development, and probably the presence of phenocopies [15, 20]. Rest of the patients had benign tumors for which they got operated and are alive without recurrence of the disease.

The MEN1 gene encodes ‘menin’ which is a nuclear scaffold protein that regulates gene transcription by altering chromatin remodeling. It functions as a tumor suppressor gene and interacts with several transcription factors like JUND, NFKB, SMAD3 etc. [19]. MEN1 gene mutations are well characterized with approximately 1330 mutations scattered throughout the entire gene comprises of mostly frameshift deletions or insertions, followed by nonsense, missense, splice-site mutations and either part or complete gene deletions [21].

Frameshift and nonsense mutations result in truncated and most probably inactive menin protein whereas splice site mutations result in incorrect splicing of the mRNA which leads to either exon skipping or addition of extra region to the final product.

Missense mutations of MEN1 are important as they result in change of crucial amino acids necessary for binding or interacting with various other molecules and proteins. This may further affect the functional activity of the menin protein or its expression levels. Studies have shown single amino acid change in the genes involved in oncogenic disorders to result in enhanced proteolytic degradation leading to reduced stability and loss of function of the mutant protein which is a common mechanism for inactivating tumor suppressor gene products [22, 12].

Menin has three nuclear localization signals (NLS) near the C terminus of the protein and the novel M561K mutation identified lies within the second NLS domain. This region of menin is postulated to have a role in interaction with transcription factors like Smad3 and CHES1 and cell cycle control proteins like ASK [15]. The patient with this mutation was a 65 years old woman who presented with episodes of fasting and post prandial hypoglycemia, frequency of which had increased over the last 8 years. The patient had one benign tumor measuring 1.5*1.2 cm located in the uncinate process of pancreas.

In the present study a missense mutation (Q192K) was identified at amino acid 192 in a 17-year-old male patient having symptoms of tremors, drowsiness, headache, generalized tonic clonic seizures for 13 months. A 2*2 cm insulinoma was localized in the pancreatic tail which was surgically removed. A previous study [13] has reported a nonsense mutation (Q192X) at this position. This region of menin interacts with proteins JunD responsible for transcription regulation, HDAC for epigenetic regulation, NMMHC II-A having a role in cell division and NM23A which plays a role in cell cycle control [15].

Apart from this, a silent mutation Q261Q identified had no change in the amino acid but the variation G > A is at the last nucleotide of exon 4/intron four boundary forming a part of conserved sequences required for splicing. This change might be resulting in incorrect splicing leading to an abnormal protein. Pathogenicity of the alteration was evaluated by Mutation Taster software which predicted the change to be disease causing (probability =1; indicating high security of the prediction).

An accurate splicing mechanism is required for removal of introns and forming the mature RNA which normally takes place by a large spliceosome complex that includes small nuclear ribonucleoprotein particles (snRNP) and a number of other proteins. This spliceosomal complex assembly requires the presence of conserved recognition sequences in the pre-mRNA for silencers, enhancers etc. These sequences may be present in the exons as well as introns. Alterations in any of these conserved sequences or elements may severely impair pre-mRNA splicing and gene expression. Various nonsense, missense and even silent mutations can inactivate genes by impairing the efficiency of splicing machinery thereby resulting in diseased phenotype [23].

Of the known polymorphisms identified in the present study, D418D (rs2071313) was the only one absent in the controls whereas H433H (rs540012) and A541T (rs2959656) were present in all the controls indicating that these polymorphisms may not be associated with increased tumor risk. However, the status of the polymorphism A541T is controversial as several studies report this to be a mutation associated with parathyroid tumors whereas others have reported this to be a non-pathogenic polymorphism present in pancreatic tumors [24–26].

Several reports also document intronic variations affecting mRNA splicing and leading to mild disease phenotypes with low penetrance [27, 28]. Two novel intronic variations c.445-44G → A and c.913-42G → C identified in the present study were absent in the control individuals suggesting their probable association with the disease. However, this needs further validation through more mutational and functional studies.

## Conclusions

In conclusion, we report the clinical and genetic findings in 17 patients with the rare condition of sporadic insulinomas. The significance of this study is the identification of three novel exonic and two novel intronic variations. The outcome of the study considerably extends the growing list of pathogenic MEN1 mutations providing a consequential insight into phenotypic heterogeneity and in the expression of individual mutations. The new mutations identified seem to confer a role in defective binding of the menin protein to specific molecules involved in a variety of genetic and epigenetic mechanisms thereby resulting in disease pathology.
